# Scaling up the Med-South Lifestyle Program in public health settings to reduce chronic disease risk: a hybrid implementation-effectiveness study

**DOI:** 10.3389/fpubh.2025.1564567

**Published:** 2025-06-05

**Authors:** Carmen D. Samuel-Hodge, Lisa Pham, Kiira Lyons, Lindy B. Draeger, Li Jiang, Feng-Chang Lin, Rachel Ram, Jennifer Leeman

**Affiliations:** ^1^Department of Nutrition, Gillings School of Global Public Health, University of North Carolina at Chapel Hill, Chapel Hill, NC, United States; ^2^Center for Health Promotion and Disease Prevention, University of North Carolina at Chapel Hill, Chapel Hill, NC, United States; ^3^Department of Biostatistics, Gillings School of Global Public Health, University of North Carolina at Chapel Hill, Chapel Hill, NC, United States; ^4^School of Nursing, University of North Carolina at Chapel Hill, Chapel Hill, NC, United States

**Keywords:** lifestyle intervention, implementation, prevention and control, COVID-19, Federally qualified health centers

## Abstract

**Introduction:**

Major disparities persist in heart disease, diabetes, and obesity, with rates highest among those living in the southeastern and central parts of the US. Intervening to improve lifestyle behaviors represents an opportunity to address health inequities. Although the scientific rationale for lifestyle interventions is robust, evidence is limited on how to implement these interventions at scale.

**Methods:**

Using a type 3 hybrid implementation-effectiveness design, we evaluated a statewide scale-up trial implementing the Med-South Lifestyle Program in mostly rural community health centers and health departments across North Carolina, in the southeastern US. Implementation outcomes were measured at the site level and program effectiveness outcomes were assessed by physiologic and behavioral changes at the participant level. Descriptive statistics and paired *t*-tests comprised our statistical analyses.

**Results:**

We invited 200 public health sites to participate in the study and 28 (14%) expressed interest. Among those expressing interest, 21 (75%) signed a Memorandum of Agreement. The statewide scale-up resulted in the enrollment of 95% (19/20) of the proposed sites−13 health departments (68%) and six community health centers. The majority of the 235 study participants who started the program were adults self-identifying as non-Hispanic White (45%) or non-Hispanic Black (37%); 11% identified as Hispanic and 5% as American Indian. Most participants were female (88%), with a mean age of 51 years, and educational attainment of a 2- or 4-year college degree (57%). Implementation outcomes included 17 sites (89%) retained throughout the study and a 79% participant retention rate. Program uptake was high, with 87% of planned counseling sessions and 83% of follow-up calls completed. For our effectiveness outcomes we observed small but statistically significant changes in weight of −2.3 lbs. Similarly, systolic but not diastolic blood pressure was reduced significantly (−2.3 mm Hg). There was a significant increase in the mean weekly intakes of nuts and healthy fats, improved daily fruit-vegetable-bean scores, and a decrease in daily sugar-sweetened beverage intake. For sedentary behaviors, daily sitting time was significantly reduced.

**Conclusions:**

These results show successfully adapted implementation and delivery approaches to fit Med-South into the context of public health settings during the COVID-19 pandemic.

**Trial Registration:**

ClinicalTrials.gov: NCT05067816, October 5, 2021.

## 1 Introduction

In the US, major disparities persist in heart disease and stroke (1), diabetes (2–4), obesity (5–7), and premature mortality (8), based on geography, race and ethnicity (9), and socioeconomic status (SES) (10). Adverse health outcomes are highest in the southeastern and central parts of the US (3–5, 11), with the highest stroke rates occurring in the southeastern US, including the coastal plain of North Carolina (NC) and other states in the ‘buckle of the stroke belt' (12). Within this geographic context, rates are highest among those self-identifying as African Americans (9), Native Americans (13), and those of lower SES (14). Residents of the southeastern US typically consume fewer fruits and vegetables (14–18) and engage in less leisure-time physical activity (16, 19, 20), compared to those living in other parts of the country. Improving these modifiable lifestyle behaviors among high-risk individuals represents an opportunity to reduce health disparities and yet lifestyle interventions are not reaching those who need them most (21, 22).

Although the scientific rationale for implementing lifestyle interventions is robust (23, 24), evidence is limited on how to scale up these interventions in clinical and public health settings, with scale up defined as “rolling out a successful local program to regional, national, or international levels” (25). Intervening to change lifestyle behaviors is difficult, and scale-up needs to address factors at the level of the patient (e.g., low literacy and lack of transportation to attend counseling sessions), the provider (e.g., lack of time and knowledge to provide counseling), the healthcare system (e.g., systems not in place to fund, coordinate, monitor, and continuously improve services), and the community (e.g., lack of access to healthy foods and places to be physically active). To be successful, the strategies used to scale up lifestyle interventions need to target these multilevel factors and build capacity to both deliver and implement interventions (26). Scale-up strategies need to build staff-level capacity to deliver lifestyle interventions, including training on current guidelines for dietary intake and physical activity and on how to counsel individuals to change those behaviors. In addition, scale-up strategies need to build setting-level capacity to implement lifestyle interventions and sustain them over time. To address patient and community-level barriers, scale-up strategies need to accommodate variations in lifestyle behaviors across differences in cultures, income levels, and the local community environments.

More than 10 years of prior Med-South research in four studies (27–30) using multiple delivery formats in public health, primary care, and community settings have brought us to this implementation research focused on statewide scale-up. The Med-South Lifestyle Program (Med-South) is a behavioral lifestyle intervention that promotes a Mediterranean dietary pattern adapted to the food culture of the southeastern US and uses evidence-based behavioral approaches to facilitate changes in dietary and physical activity habits. The Med-South dietary pattern is highly concordant with those associated with reduced risk for many chronic diseases and all-cause mortality (31) and is consistent with the latest USDA Dietary Guidelines (22, 32). Med-South has been delivered by health professionals and community health workers in formats that include individual in-person, group- and web-based, and hybrid formats of in-person and phone-based counseling sessions. The first two Med-South studies (27, 28) demonstrated the program's effectiveness in reducing coronary heart disease risk by improving blood pressure, blood lipids, and lifestyle behaviors among patients in family medicine practices and community-based residents. With demonstrated program effectiveness, the next two studies (29, 30) focused on implementation strategies in public health settings using hybrid effectiveness-implementation study designs. In these studies, we selected and tailored strategies to implement Med-South in a small number of Community Health Centers (CHCs) and health departments (HDs), both of which have broad reach to at-risk populations. Guided by the Barker et al. framework for scaling interventions (33), we engaged key partners in the iterative design and testing of implementation strategies. To begin planning for scale-up we consulted with our community advisory board and engaged representatives from state-level CHCs and HDs and other community organizations to adapt the intervention, tailor implementation strategies, and plan the pilot testing of Med-South in two counties. We used the Expert Recommendations for Implementing Change (ERIC) (34) to guide the organization of our implementation strategies (including who enacted the strategy, its central purpose, and the specific activities used in its implementation). Successful implementation of Med-South in these rural public health settings set the stage for tailoring these implementation strategies for the current study focused on statewide scale-up (35). This tailoring of implementation strategies for statewide scale-up involved (1) reducing the high level of research team involvement, which would not be feasible for scale-up or program sustainability, and (2) selecting and tailoring new strategies to address barriers and leverage facilitators (35).

In this paper, we report findings from the scale up of Med-South in North Carolina (NC), where approximately 4 million people, or about 40% of the population, live in one of the state's 78 rural counties (36).

We describe the implementation and effectiveness outcomes of our statewide scale-up trial in mostly rural CHCs and HDs across North Carolina. Two types of trial outcomes are reported—implementation outcomes at the site level and program effectiveness outcomes as measured by physiologic and behavioral changes at the participant level.

## 2 Methods

### 2.1 Design

This study used a Type 3 hybrid effectiveness-implementation pre-test/post-test trial design (37). Hybrid designs focus on both the effectiveness of a program (how well it works to improve the health of participants) and how a program is implemented or put into place in real-world practice settings. In a type 3 design, the main focus or primary aim is to evaluate the impact of implementation strategies, with program effectiveness as a secondary aim (35). While we expected our effectiveness outcomes to be similar to previous studies using the Med-South Program, our focus was on how best to place this program in public health settings. In our prior study (30) using the same design, we focused on developing and testing implementation strategies to integrate Med-South in two counties. In the current study we tailored those strategies for scale-up and tested the roll-out of Med-South statewide. We proposed to recruit and enroll 20 sites (10 Federally Qualified Health Centers (FQHCs)/community health centers (CHCs) and 10 local HDs) with 20 participants per site for a total of 400 participants.

This study was approved and monitored by the University of North Carolina Non-Biomedical Institutional Review Board (IRB). In July 2021 the Med-South Lifestyle Program implementation phase of the study was approved; direct interaction with study participants ended in December 2023. We recruited and enrolled staff from CHCs and local HDs, and site staff referred potential participants to University staff for screening, consent and enrollment. Site staff provided written informed consent and all participants provided verbal informed consent.

### 2.2 Med-South Lifestyle Program

Med-South is a 4-month behavioral lifestyle intervention targeting dietary and physical activity behaviors. Table 1 outlines the 4 monthly counseling visits and 3 follow-up calls with a description of the program's content. The recommended dietary pattern is a Mediterranean dietary pattern adapted for the southeastern US food culture, with a focus on affordable and familiar foods such as peanuts or peanut butter, vegetable oils, and modified recipes for traditional southern foods such as hush puppies, collard greens, and barbeque. Med-South dietary goals include nuts/nut butters and beans 3 times weekly, at least 7 servings daily of fruits and vegetables, and < 1 sugar-sweetened beverage daily. Physical activity goals align with the recommendation that US adults engage in at least 150 min of physical activity per week (38). Counseling visits were delivered as individual in-person sessions (sessions 1 and 4) and via phone or virtual format (e.g., Zoom) for sessions 2 and 3. Optional session content included information on addressing barriers to medication adherence. Additionally, counselors could make a 4th follow-up call after the last program visit. At the first counseling visit, participants received a program manual, a cookbook, and a local resource guide with information on where to find healthy food options, places to be physically active, wellness classes, and medication assistance. Each counseling session begins with an assessment of current eating habits specific to the session topic. This assessment allows the counselor to tailor the session content to align with what participants want to know and which behaviors they want to address first among those needing the most improvements. The counselor and participant work together to set no more than 2 achievable goals and plan actions needed to reach their goals. While goals are set for the month, at the follow-up calls participants could modify or keep their goals based on their level of progress. One month after each of the first 3 monthly sessions, counselors entered data in REDCap on progress made by participants in reaching each goal set.

**Table 1:** Med-South Lifestyle Program content and contacts.^a^

**Program contacts**	**Program content^b^**
Counseling visit 1	**Nuts, oils, dressings and spreads** ∎ Types of fats, healthy fats, and daily intake recommendation ∎ Ways to fit healthy fats into eating plan
Counseling visit 2	**Vegetables, fruits, whole grains and beans** ∎ Types of fruits and vegetables and how much to have daily ∎ Importance of eating whole grains and beans and how often to eat
Counseling visit 3	**Drinks, desserts, snacks, and eating out and salt** ∎ Reasons to limit sugary drinks and what to drink instead ∎ Choosing healthy desserts, sweets, and snacks ∎ Strategies for making healthy choices when eating out
Counseling visit 4	**Fish, meat, dairy and eggs** ∎ Why fish is important, how much to have, and how to prepare ∎ What to know about red meats, processed meats, dairy products, and eggs, and how much to have
Counseling visits 1–4 (additional content)	**Physical activity** ∎ Types of aerobic and muscle-strengthening physical activity and how much is recommended ∎ Safety when walking ∎ Chair exercises for strength and flexibility **Healthy eating: additional information** ∎ Serving sizes, meal prepping, kitchen tips and tricks, best oils for cooking ∎ Eating healthy on a budget
Follow-up calls 1–3 (optional 4th call)	∎ Check-in on goal progress (successes and challenges related to the topic(s) covered) ∎ Check-in on referrals (actions taken or barriers to following through or receiving services) ∎ Reminder or scheduling of next counseling session
Optional content	**Taking medications** ∎ What you should know ∎ Reasons for not taking medication and ways to address ∎ Local pharmacies

^a^Monthly counseling visits (planned for 45–60 min duration) were delivered as individual in-person visits for sessions 1 and 4 because weight and blood pressure are measured, and via phone or virtual formats for sessions 2 and 3. Follow-up calls with planned duration of 10–15 min were scheduled for 10–14 days (about 2 weeks) after each of the first 3 counseling visits. An optional follow-up call after the 4th counseling visit may be provided by counselors to close out their engagement with participants.

^b^Standard content for each session included a brief assessment of current eating habits, sharing of background information, tips for reaching goals, behavioral goal setting and action planning, and referral to community resources if indicated.

### 2.3 Implementation strategies

Implementation strategies tested in our prior research (35) were tailored for use in the current research. The full set of implementation strategies used in this study is listed in Table 2, using terminology drawn from the Expert Recommendations for Implementing Change (ERIC) compendium of strategies (34). Each participating site was asked to identify staff to deliver the Med-South Program (counselors) and staff to form an implementation team. At each site, implementation teams comprised two to five staff, which typically included the counselors and a range of other staff (e.g., quality improvement, supervisory, or administrative staff). Teams were asked to create a team charter, meet monthly, and work together to integrate Med-South in clinic workflow. Counselors and members of the implementation team participated in trainings which included 2 self-guided online modules on nutrition and physical activity, completed by counselors, and a series of four 2-h web-based live trainings on program delivery and implementation, completed by both counselors and implementation teams. To comply with IRB requirements for staff engaging with study participants, the designated counselors also completed CITI trainings on Human Subjects Research and Good Clinical Practice (39). Additional strategies included distribution of educational materials (participant manuals and cookbooks) and monthly technical assistance calls.

**Table 2:** Implementation strategies.

**Strategy (ERIC)^a^**	**Activities**
Organize site implementation team meetings	∎ Site implementation team creates a team charter, meets monthly, and integrates Med-South into site's health service workflow
Provide ongoing training	∎ Counselors complete self-guided online modules on dietary and physical activity guidelines ∎ Counselors attend web-conferences on how to deliver Med South (4 h on intervention delivery) ∎ Implementation team attends web-conferences on how to implement Med South (4 h on implementation)
Distribute educational materials	∎ Research team provides sites with participant manual, cookbooks, and an inventory of community resources
Develop and implement tools for quality monitoring	∎ Counselors enter intervention delivery data into REDCap^b^ (a data capture and management system)
Centralize technical assistance	∎ Research team coaches implementation team on Med South delivery and implementation during monthly technical assistance phone calls

^a^ERIC (Expert Recommendations for Implementing Change) (34).

^b^REDCap (Research Electronic Data Capture).

### 2.4 Site recruitment

The study team engaged both a Community Advisory Board (CAB) and study-specific workgroup for advice on site recruitment. CAB and workgroup members included representatives of governmental public health organizations (e.g., local health departments, community health centers, rural health groups), community based organizations, Area Health Education Centers (AHEC), and other health-related professionals. Sites were recruited in three cohorts using multiple recruitment methods, including referral from pilot study sites, listserv emails, and follow-up with statewide online survey respondents from the first phase of the project (35). Once a site expressed interest in joining the study, staff scheduled brief introductory phone calls to share additional information and answer questions. Sites were required to have two to four dedicated staff as members for an implementation team, including one staff member as the counselor delivering Med-South. Sites with a Spanish-English bilingual counselor could recruit Spanish-speaking adults. Once a site determined it had adequate staff and resources for joining the study, a Memorandum of Agreement outlining responsibilities was signed. Sites included both local HDs and CHCs. Given that the timing of site recruitment began in 2021 during the COVID-19 pandemic, we recruited sites in three cohorts because of recruitment challenges stemming from sites being overwhelmed responding to the pandemic. Sites were reimbursed for their roles in recruitment of participants and program delivery. Reimbursements include $5,000 for staff training time and use of office space for program delivery, and $75 for each hour of counseling (estimated at 6 h total (4 session + 3 calls) per participant).

### 2.5 Participant recruitment

Upon training completion, site staff recruited participants through posting flyers, community outreach, word-of-mouth, and provider referrals. Interested individuals were referred to research staff at UNC for phone screening and consent. Inclusion criteria included: 18–80 years old, English or Spanish speaking, able to make decisions about dietary intake, no advanced kidney disease (estimated creatinine clearance < 30 ml/min), and no diagnosis of malignancy or cancer. After obtaining informed consent, research staff administered a baseline survey, including questions about current eating and physical activity habits, food security, medication adherence, and other health and demographic characteristics. Participants were given a $40 incentive for completing baseline and follow-up phone surveys.

### 2.6 Data collection

We measured both implementation and effectiveness outcomes as described below. Study data were collected and managed using REDCap (Research Electronic Data Capture) electronic data capture tools hosted at the University of North Carolina, Chapel Hill. REDCap is a secure, web-based software platform designed to support data capture for research studies by providing (1) an intuitive interface for validated data capture; (2) audit trails for tracking data manipulation and export procedures; (3) automated export procedures for seamless data downloads to common statistical packages; and (4) procedures for data integration and interoperability with external sources (40–42). Research staff phone-administered all participant data collection surveys (baseline and post-intervention follow-up with program acceptability). Participants received $40 each for the baseline and follow-up data collection survey.

Our implementation and program effectiveness data and outcomes are described below. Both research and site staff collected data for implementation and effectiveness outcomes. For implementation data collection, counselors at each site received training on entering program delivery fidelity data into REDCap, while research staff were responsible for collecting data on adoption and reach via a tracking log maintained by the study coordinator. To determine program effectiveness, site staff collected participant physical measures of weight and blood pressure at the first and last program counseling visit. Research staff collected survey data on dietary and physical activity behaviors pre- and post-intervention.

#### 2.6.1 Implementation outcomes

Measurement of implementation outcomes was guided by Proctor's outcomes framework (43). Included in this report are implementation outcomes related to program adoption, reach, delivery fidelity, and acceptability. We will report separately on implementation costs and key informant interview findings on barriers and facilitators to successful scale-up. Adoption was operationalized as the number, proportion, and characteristics of eligible sites invited, enrolled, and retained in the study. Reach was operationalized as the number and demographics of individuals referred for enrollment, enrolled, and retained. Data on intervention delivery fidelity entered in REDCap included session attendance, goals set, referrals made to community resources, follow-up calls completed, and duration of sessions/follow-up calls. Research staff administered a survey on program acceptability as part of the post-intervention survey, which included questions about barriers to attendance; satisfaction with program delivery format, program materials, counseling experience, and health outcomes; and confidence in maintaining behavior changes.

#### 2.6.2 Effectiveness outcomes

We used validated measures to assess self-reported dietary and physical activity behaviors. Dietary intake data collection included brief measures of fruit, vegetable, and fiber (44), sugar-sweetened beverages (45), and intake of nuts and nut butters (46). Physical activity behaviors were measured with the modified RESIDential Environment (RESIDE) survey (47), and sedentary behaviors were measured with a sitting behavior item from the Global Physical Activity Questionnaire (GPAQ) (48). We also measured food security (49) and collected general health and demographic information. Weight was measured using SECA scales (model 874, Seca, Hanover, MD) and reported as an average of two measurements. Blood pressure was assessed with an automated Omron (HEM-907XL, Omron Healthcare, Bannockburn, IL) monitor with reporting as the average of two measurements taken at 1-min intervals, following a 5-min rest period.

### 2.7 Statistical analysis

Baseline demographic data were summarized as mean (standard deviation) for continuous variables or number (%) for categorical variables. Pre-post changes at 4 months were compared using paired *t*-tests for continuous outcomes. Comparison of demographic variables was conducted by logistic regression or chi-squared test. Analysis of variance (ANOVA) was utilized to assess differences among groups for continuous variables, while chi-squared tests were employed for categorical variables. Given our type 3 hybrid implementation-effectiveness design where intervention effectiveness is a secondary outcome, we did not account for missing values by utilizing imputation methods but provide data on participants lost to follow-up. All analyses were conducted using R Statistical Software [v4.2.2; (66)]. The statistical significance was set at *p*-value ≤ 0.05.

## 3 Results

Our statewide scale-up resulted in the recruitment of 19 of the 20 sites (95%) we proposed to recruit, with more local HDs (*n* = 13) enrolling than CHCs (*n* = 6). Sites were recruited over an 18-month period in 3 cohorts of 6–7 sites each. Table 3 shows our implementation outcomes related to adoption—our recruitment, enrollment, and retention of study sites. We invited 200 sites to participate in the study and 28 (14%) expressed interest. Among those expressing interest, 21 (75%) signed a Memorandum of Agreement (MOA) and 19 (90%) of those completed the required Med-South implementation and delivery training.

**Table 3:** Implementation and delivery effectiveness outcomes.

**Implementation variables**	**Mean (SD) or *N* (%)^a^**
**Site recruitment, enrollment, and retention**	
Sites expressing interest/sites invited (*n* = 200), %	28 (14)
Sites expressing interest with signed MOA,^a^ %	21 (75)
Sites with signed MOA completing training, %	19 (90.5)
Trained sites starting program delivery, %	18 (94.7)
**Participant recruitment and enrollment**	
Referred adults (*n* = 551) completing screening, %	311 (56.4)
Consented participants/Screened, %	301 (96.8)
Enrolled participants/Consented, %	299 (99.3)
**Program Delivery (*****n*** = **235)**	
Proportion of total planned counseling visits (*n* = 940) attended, %	816 (86.8)
Proportion of total planned phone follow-up calls^b^ (*n* = 705) completed, %	587 (83.3)
Counseling visits completed/participant, mean (SD)	3.47 (0.99)
**Contact duration in minutes, mean (SD)**	
Counseling visits	52.0 (18.3)
Brief phone follow-up between counseling sessions	17.0 (12.6)
Mean number of goals set/counseling session	2.1 (0.5)
**Proportion of dietary goals set at counseling**	991 (70.6)
**visits 1–3**^b^ **that were met by the following**	
**visit, % (*****n*** = **1404)**	
Session 1 goals (*n* = 523)	394 (75.3)
Session 2 goals met (*n* = 513)	330 (64.3)
Session 3 goals met (*n* = 368)	267 (72.6)
Proportion of physical activity goals set at counseling visits 1–3^b^ that were met by the following visit, % (*n* = 342)	183 (53.5)
Mean number of referrals made/counseling session	2.6 (2.8)
**Program Acceptability (*****n*** = **147)**	
**Barriers to session attendance, %**	
COVID-19	25 (17.0)
Work schedule	37 (25.2)
Family responsibilities	29 (19.7)
Personal health/illness	23 (15.6)
Transportation	4 (2.7)
Schedule of counseling visit	18 (12.2)
Loss of interest or motivation	12 (8.2)
**Length of monthly counseling sessions, %**	
Too short	7 (4.8)
Just about right	139 (94.6)
Too long	1 (0.7)
**Total number of sessions and follow-up calls, %**	
Too few	11 (7.5)
Just about right	135 (91.8)
Too many	1 (0.7)
**Comfort with meeting health counselor in-person at first**	
**and last visit, %**	
Not at all comfortable	2 (1.4)
Somewhat comfortable	6 (4.1)
Very comfortable	139 (94.6)
**Satisfaction**^c^ **with program components, mean (SD)**	
Med-South manual	4.9 (0.5)
Referrals to local resources	4.9 (0.4)
Change in weight	4.1 (1.1)
Change in blood pressure	4.6 (0.8)
Change in overall health	4.5 (0.7)
Visits with health counselor	4.8 (0.5)
**Confidence**^c^ **in behavior change maintenance, mean (SD)**	
Continue to eat healthy foods	4.8 (0.5)
Continue to be physically active	4.5 (0.8)
Continue to take blood pressure meds	4.9 (0.5)
Continue to take cholesterol meds	4.9 (0.2)
Maintain the weight lost	4.4 (0.8)
Remain a non-smoker	4.8 (0.7)

^a^Memorandum of agreement.

^b^There was no planned follow-up to counseling session 4, but some sites made optional follow-up calls (n = 92).

^c^5-point Likert scale with 1 = not at all satisfied/confident and 5 = very satisfied/confident.

Thirteen of the 19 enrolled sites (68%) were located in NC counties designated as rural (counties with an average population density of <250 people/mi^2^) (50). Statewide, 78% of NC counties are designated as rural using this definition, and we succeeded in enrolling public health sites across the entire state. Eighteen of the 19 enrolled sites (95%) delivered Med-South to their participants, and we retained 17 sites (89%) throughout the program implementation and delivery phase. One health department lost its trained counseling staff after participants were recruited and had to withdraw from the study, and we lost a CHC after starting program delivery when it came under new management.

**Figure 1:**
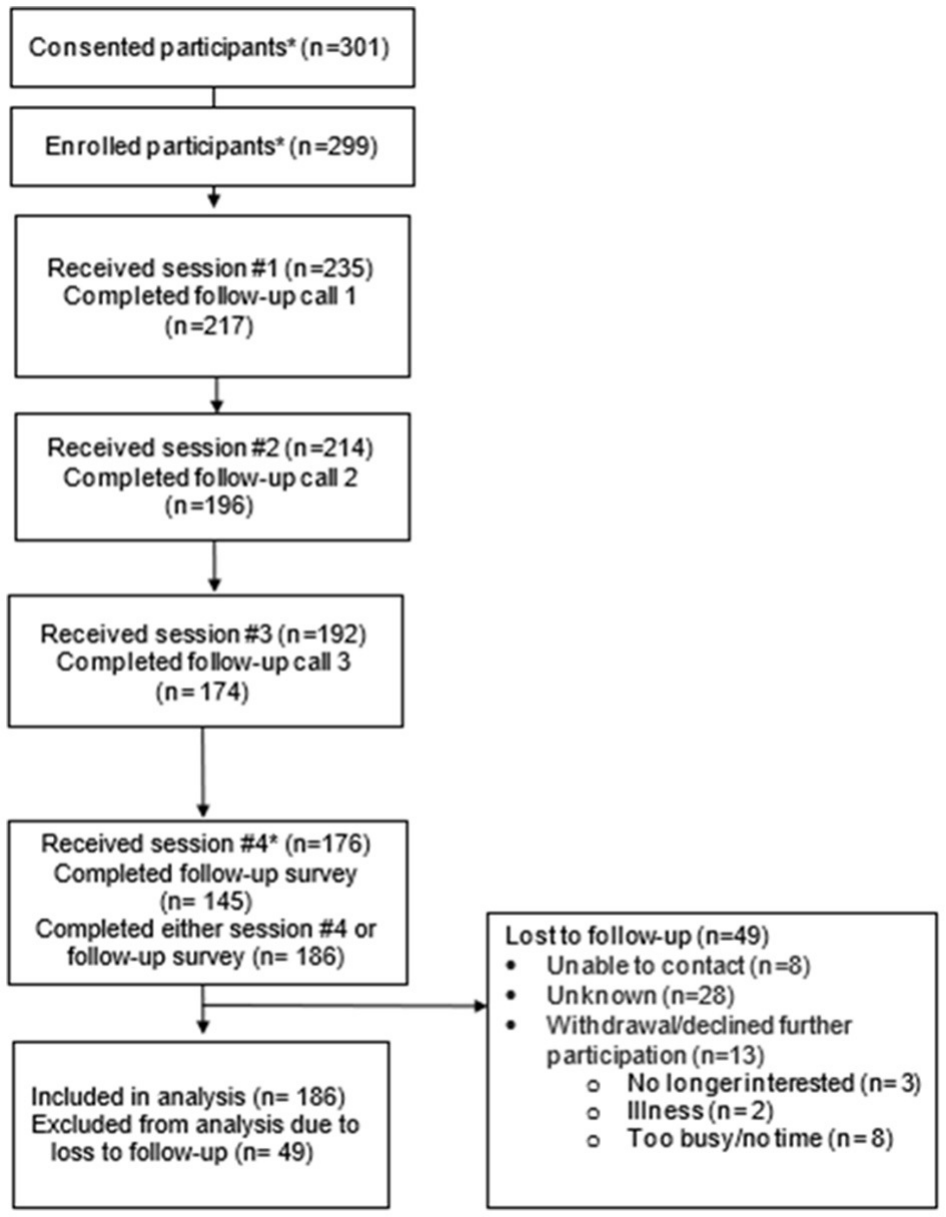
Participant flow diagram.

For reach, Table 3 includes our participant recruitment and enrollment outcomes; Figure 1 shows the flow of participants through the study, and Table 4 describes participants by site type (HDs and CHCs). We proposed recruiting up to 400 participants (20 sites with 20 participants each) but because of the COVID-19 pandemic, we reduced our total enrollment goal to 300 after our pilot phase showed that 15 per site would be more feasible. Sites referred 551 adults to UNC study staff for screening, consent, and enrollment. Among those referred, 311 (56%) completed eligibility screening, 301 (97%) of those screened were consented, and 299 (99%) of those consented were enrolled (e.g., completed the baseline survey). Once participants were enrolled by UNC staff, their information was shared with sites to begin program delivery. Among enrolled participants, 235 (79%) began the program (completed the first session), 214 (91%) completed the second session, 192 (82%) completed the third, and 176 (75%) completed the fourth session (Figure 1). Our participant retention rate was 79% with 49 participants lost to follow-up. Reasons for participant attrition were mostly unknown, with about 26% (*n* = 13) withdrawing because they were too busy, no longer interested, or ill. Participants who were lost to follow-up did not differ significantly by demographic characteristics when compared to those providing follow-up data.

**Table 4:** Participant characteristics by public health site type.

**Characteristic**	**Health department (*n* = 161)**	**Community health center (*n* = 74)**	**Total (*n* = 235)**
	***N*** **(%) or Mean (SD)**	***N*** **(%) or Mean (SD)**	***N*** **(%) or Mean (SD)**
**Race/Ethnicity, %**			
American Indian	12 (7.5)	1 (1.4)	13 (5.5)
Hispanic	11 (6.8)	14 (18.9)	25 (10.6)
Non-Hispanic Black	68 (42.2)	20 (27.0)	88 (37.4)
Non-Hispanic White	67 (41.6)	38 (51.4)	105 (44.7)
**Participant type, %**			
Community member	98 (60.9)	73 (98.6)	171 (72.8)
Employee	63 (39.1)	1 (1.4)	64 (27.2)
Female, %	151 (93.8)	57 (77.0)	208 (88.5)
Mean Age, year	50.5 (13.6)	51.4 (12.4)	50.8 (13.2)
**Education, %**			
High school diploma or less	30 (18.6)	33 (44.6)	63 (26.8)
Some college	23 (14.3)	15 (20.3)	38 (16.2)
College degree (2-year or higher)	108 (67.1)	26 (35.1)	134 (57.0)
Currently living with a spouse or someone like a spouse or partner, %	95 (59.0)	41 (55.4)	136 (57.9)
**Physiologic and Behavioral Characteristics**			
Current Smoker, %	10 (6.2)	9 (12.2)	19 (8.1)
Diagnosed hypercholesterolemia, %	64 (39.8)	40 (54.1)	104 (44.3)
Diagnosed hypertension, %	73 (45.3)	41 (55.4)	114 (48.5)
Diagnosed diabetes, %	24 (14.9)	30 (40.5)	54 (23.0)
Mean Systolic blood pressure, mm Hg (*n* = 233)	128 (15.7)	128 (17.4)	128 (16.2)
Mean Diastolic blood pressure, mm Hg (*n* = 233)	79.9 (10.2)	80.3 (10.4)	80.0 (10.3)
Mean Weight, lbs. (*n* = 234),	203 (49.5)	232 (53.4)	212 (52.4)
Mean BMI, kg/m^2^ (*n* = 233)	34.3 (7.61)	38.1 (8.59)	35.5 (8.11)
Mean Physical activity, min/week	203 (222)	174 (372)	194 (277)
**Sedentary Behaviors**			
Mean Sitting time, h/day	6.89 (3.14)	5.86 (2.98)	6.57 (3.12)
Currently tracking daily step count, %, (*n* = 88)	75 (46.6%)	13 (17.6%)	88 (37.4%)
Mean Fruit & vegetable servings/day	3.96 (1.40)	4.09 (1.56)	4.00 (1.45)
Mean Daily fruit, vegetable, bean score^a^	14.4(5.05)	14.6 (5.07)	14.5 (5.05)
Healthy fats, nuts servings/week^b^ (*n* = 234)	1.99 (0.92)	1.90 (0.90)	1.96 (0.91)
Mean Sugar sweetened beverages/day	0.61 (0.87)	0.78 (1.40)	0.66 (1.07)
**Household Food Insecurity**			
Low Food Security, %	21 (13.0)	18 (24.3)	39 (16.6)
Very Low Food Security, %	7 (4.3)	7 (9.5)	14 (6.0)
**Medication Adherence Subscales, mean scores** ^c^			
Behavior (*n* = 178)	22.9 (3.01)	22.5 (2.53)	22.8 (2.85)
Health beliefs (*n* = 176)	7.21 (2.52)	7.65 (2.17)	7.37 (2.41)
Inconvenience/forgetfulness (*n* = 179)	12.4 (2.49)	11.4 (2.77)	12.1 (2.63)

^a^Minimum = 2; maximum = 29.

^b^A score of 1 = 0–1 servings/week.; 2 = 2 servings/week.; 3 = 3 or more servings/week.

^c^Higher scores represent greater adherence barriers. Score ranges – behavior subscale = 5–25; health beliefs = 4–20); inconvenience/forgetfulness = 3–15.

Participant characteristics for those that started the program (*n* = 235) are described by site type in Table 4. When we compared the participants who enrolled but did not start the program, we found no significant differences in demographic characteristics. Overall, most Med-South participants (68%) were at local HDs with the majority (73%) recruited as community members, with the remaining recruited as site employees. Individuals self-identifying as non-Hispanic White (45%) or non-Hispanic Black (37%) comprised the majority of participants; 11% identified as Hispanic and 5% as American Indian. Most participants were female (88%), with a mean age of 51 years, educational attainment of a 2- or 4-year college degree (57%), and currently living with a spouse or partner (58%). In comparing demographic characteristics by site type, Table 3 shows greater participation of American Indians, non-Hispanic Blacks, and employees at HDs compared to CHCs. Participants at HDs also had higher educational attainment compared to those at CHCs.

Overall, reported chronic conditions included 48% diagnosed with hypertension, 44% with hypercholesterolemia, and 23% diagnosed with diabetes; 8% reported being current smokers. At baseline, the average blood pressure was 128 mm Hg systolic and 80 mm Hg diastolic pressure; mean weight was 212 lbs., and BMI 35.3 kg/m^2^. Lifestyle behaviors assessed included physical activity and sedentary behaviors, and dietary intake. Participants reported an average of 194 min of weekly physical activity with about 37% tracking daily step counts, and average sitting time of over 6 h/day. Self-reported dietary intake included mean intake of 4 fruit and vegetable servings/day; a daily fruit, vegetable and fiber score of 14.5; a score of 1.96 or nearly 2 servings of healthy fats and nuts per week; and less than one sugar-sweetened beverage daily. Our assessment of food security showed over 16% with low food security and 6% with very low food security (categories of food insecurity). For participants with prescribed medications, we measured adherence behaviors, beliefs, and inconvenience or forgetfulness. Mean subscale scores show that perceived barriers were highest for adherence behaviors (e.g., barriers to taking medicines as prescribed) and inconvenience/forgetfulness. If we look at these participant characteristics by site type, at CHCs we observed a higher proportion of participants with diagnosed diabetes, fewer participants who track their daily steps, and a larger proportion of participants with food insecurity.

Our program delivery outcomes are included in Table 3 with results for program delivery fidelity (delivery of the program as intended) and participant enactment fidelity (participants' use of skills targeted by the program) (51). For the 235 participants who started the program, there were 940 planned counseling sessions and of these 816 (87%) were attended. Likewise, there were 705 planned follow-up calls of which 587 (83%) were completed. On average participants completed 3.5 of the 4 planned counseling visits with a mean duration of 52 min. For the three planned follow-up calls, 2.5 were completed on average with a mean duration of 17 min. Although a 4th follow-up call was not required, some counselors decided to follow up with participants after the last counseling session to have a brief conversation about progress made with the goals set and close out their counseling. These optional calls were made to 39% of participants.

Med-South counseling sessions are designed for participants to achieve their behavioral goals through skill-building in goal setting and action planning. Participants are encouraged to set no more than 2 realistic and achievable goals per session. Results of our participant enactment fidelity shows that at each counseling session, participants set 2 goals on average. Moreover, if we assess skills in goal setting and action planning by the proportion of dietary goals set that were met by the next counseling visit, we observe that overall, 70% (991/1,404) of the dietary goals set at the first 3 counseling visits were met. Among the physical activity goals set, over half (53%) were met at the following visit. Counselors also referred participants to community resources if additional support was needed to make the desired behavior changes. These referrals averaged over 2 per counseling session.

We also assess participants' views of the program with a short acceptability survey (see Table 3). Perceived barriers to program participation were minimal, with work schedules perceived as the top barrier by 25% of respondents. Most participants (92%−94%) felt the length of sessions and the number of sessions were “just about right.” Likewise, most (95%) felt comfortable meeting with the health counselor in-person. Satisfaction with program components and personal health outcomes showed overall high ratings (4.1–4.9 on a 5-point scale), as did confidence in maintaining their healthy lifestyle behaviors (4.4–4.8).

Table 5 shows our Med-South Program effectiveness outcomes with pre-/post-intervention changes in weight, blood pressure, and lifestyle behaviors. Our data showed small but statistically significant changes in weight of −2.3 lbs. Similarly, systolic but not diastolic blood pressure was reduced significantly (−2.3 mm Hg). Since the mean systolic and diastolic blood pressure at baseline were < 130 and 80 mm Hg, respectively, we assessed changes in the proportion of participants with systolic < 130 or diastolic blood pressure < 80 mm Hg. At post-intervention there was nearly a 9% increase in the proportion of participants with a systolic blood pressure < 130, and a 9% decrease in the proportion with a diastolic blood pressure < 80, with both changes deemed non-statistically significant. Our lifestyle changes in dietary and physical activity behaviors were mostly statistically significant except for marginally significant changes in fruit and vegetable intake and weekly physical activity minutes. We observed a significant increase in the mean weekly intakes of nuts and healthy fats, improved daily fruit-vegetable-bean scores, and a decrease in daily sugar-sweetened beverage intake. For sedentary behaviors we observed a significant reduction in the self-reported daily sitting time. Additionally, there were no significant associations of demographic characteristics (e.g., age, sex, race/ethnicity, and education) with dietary behavioral outcomes. Neither was food insecurity associated with dietary behavior changes. Because of our small sample size of community health center sites and participants, we did not compare our effectiveness outcomes by site type.

**Table 5:** Program effectiveness outcomes.

**4-Month outcome**	***N* ^a^**	**Pre-intervention mean (SD)**	**Post-intervention mean (SD)**	**Mean change (SD)**	***P*-value**
Weight, lb.	173	211.4 (55.5)	209.1 (54.3)	−2.3 (9.7)	0.002
Systolic blood pressure (SBP)	158	128.6 (15.6)	126.3 (15.3)	−2.3 (14.1)	0.04
Diastolic blood pressure (DBP)	158	80.0 (10.5)	79.4 (10.9)	−0.56 (10.54)	0.51
**Proportion at goal** ^a^	158				
SBP (< 130), %		53.2	62.0	8.8	0.11
DBP (< 80), %		48.7	39.9	−8.8	0.11
Nuts, healthy fats, servings weekly	145	1.96 (0.92)	2.26 (0.85)	0.30 (1.04)	0.0006
Fruit & vegetable servings, daily	145	4.1 (1.5)	4.3 (1.2)	0.23 (1.46)	0.058
Fruit, vegetable, bean score, daily	145	14.8 (5.1)	15.9 (4.4)	1.1 (4.8)	0.005
Sugar-sweetened beverages, servings daily	145	0.56 (1.02)	0.34 (0.58)	−0.22 (1.04)	0.014
Physical activity, weekly minutes	145	215.3 (319.0)	287.7 (460.9)	72.4 (472.5)	0.067
**Sedentary behavior**					
Sitting time, h/day	145	6.7 (3.4)	5.9 (2.8)	−0.82 (2.96)	0.001

^a^Excludes participants with missing blood pressure values at the last intervention visit.

## 4 Discussion

In this study where we proposed scale-up of the Med-South Program to 20 public health sites, we demonstrated that our implementation strategies supported the successful scale-up of the Med-South program statewide despite the impact of the COVID-19 pandemic. Eighteen sites adopted Med-South and 17 of those sites completed program delivery with a high level of fidelity. Within the context of our study's site recruitment goal, this outcome is certainly positive, but in the larger context of the number of public health sites invited to participate, having only 14% (28/200) express interest in study participation may signal that sites perceive more barriers than benefits to this type of program implementation. In a prior study in NC local health departments (52), we found 30 of 81 sites (37%) were eligible and interested in study participation to implement a 5-month weight loss program. In contrast, only 13 of 84 health departments (15%) expressed interest in Med-South implementation. The COVID-19 pandemic certainly had a negative impact on our ability to recruit sites to implement a short program with only 7 contacts, but there are many longstanding barriers to health departments providing preventive healthcare services (e.g., financial, time, and workload constraints) (53).

Overall, Med-South participants received 87% of program counseling sessions and 83% of follow-up phone calls. Whether implementation strategies were successful at reaching participants is difficult to assess. The delivery of implementation strategies within the context of a research study introduced barriers to the recruitment process. To complete requirements of the research study, participating sites referred potential participants to the research team to complete the consent process and baseline data collection. The research team then sent lists of enrolled participants back to the HDs and CHCs to schedule the first counseling visit. As a result, only a subset of interested participants were enrolled by the research team and only a subset of those initiated the Med-South Program. Once participants initiated Med-South, most were retained. Of the 235 who started Med-South, 79% completed the program, demonstrating a high rate of retention for a lifestyle change program implemented in public health settings (24, 54, 55). Furthermore, the program demonstrated broad reach to African American and Hispanic participants (37% and 11% of participants, respectively). As is true of most lifestyle change interventions, most participants were women (88%). Reflecting on how we tailored our implementation strategies for scale-up of Med-South gives us insight into how we may further refine our approach. Our creation of site-based implementation teams and the training they received on tailoring and iteratively improving implementation strategies reduced the need for additional support from the research staff. Modifications made in our training for Med-South implementation and delivery, which included a shorter, virtual format and brief monthly technical assistance to reinforce training content, likely contributed to our positive implementation outcomes. Additional tailoring for scale-up will, however, be needed in developing and implementing simple tools for quality monitoring of program delivery, without using REDCap software.

Effectiveness outcomes were secondary for this hybrid study design but given the context in which this study was conducted, our results provide meaningful insights. One factor that may have influenced the outcomes of this study is the COVID-19 pandemic, which necessitated a change in format of our program delivery and potentially affected the type of participants who participated and their ability or motivation to make lifestyle behavior changes. From research describing how lifestyle behaviors were affected during COVID-19, we have reports of lower levels of adherence to a Mediterranean dietary pattern, worsened dietary quality, increased weight gain, and less physical activity as compared to before COVID-19 (56–58). The sample referred by sites for enrollment in this study included a larger proportion of adults (57%) with 2- to 4-year college degrees (especially among those referred by HDs) than our prior implementation study samples, where it ranged from 22 to 26% (29, 30). Moreover, the proportion of participants with diagnosed diabetes and hypertension in prior study samples was nearly twice that of this study. Despite the COVID-19 environmental context and the adaptations to delivery format, we observed program outcomes comparable to our prior studies with similar participants. In two prior studies (30, 31) mean changes in systolic blood pressure were −2.5 to −5.4 mm Hg, fruit and vegetable servings/day increased 0.8–0.9 servings/day, and physical activity 40–45 min/week, while changes in the current study were −2.3 mm Hg, 0.2 servings/day, and 72 min/week, respectively. We observed smaller improvements in fruit and vegetable intake in this study where baseline intakes were higher, but other program outcomes were similar between studies. These outcomes suggest that this person-centered and culturally-tailored approach to lifestyle behavior change fits a diverse demographic southeastern US population.

In addition to improvements in self-reported dietary behaviors, we observed positive changes in objectively measured weight and blood pressure. Though the Med-South Program is not designed to achieve clinically meaningful weight loss, and during COVID-19 there were potentially clinically significant increases in weight gain (about 2 lbs. in adults) (56), we observed a statistically significant reduction in mean weight of about the same magnitude. For blood pressure, we only observed a statistically significant reduction in mean systolic blood pressure. When compared to the pooled changes in systolic blood pressure of −1.81 mm Hg reported in the updated USPSTF review of behavioral counseling interventions to promote a healthy diet and physical activity for CVD in adults with cardiovascular risk factors, our reduction of −2.3 mm Hg is comparable.

Our implementation and effectiveness outcomes represent strengths of this study and have enhanced our understanding of scaling up Med-South statewide. There are, however, limitations of this study worth noting. First, even in a hybrid implementation-effectiveness trail, a single arm, pre-post study design confers limitations in attributing the observed program effectiveness to the intervention alone. That said, given the COVID-19 pandemic context during this study and its reported impact on lifestyle behaviors, we are more confident in outcomes being related to Med-South. Second, there is the potential of selection bias impacting our ability to generalize our findings to public health sites in the state. In a prior implementation study involving local health departments in NC (52, 59), we used an optimized probability sampling of study sites to randomly select a combination of 6 sites from 30 sites expressing interest and determined eligible. Optimization ensured the inclusion of different types of health departments (e.g., from small, medium, and large counties, with service populations representing the racial/ethnic makeup of the state, etc.). We were not able to optimize this study sample because the COVID-19 pandemic caused significant site recruitment challenges. Third, our loss-to-follow-up and missing data for blood pressure (33% missing) and survey measures of behavioral outcomes (38% missing) resulted in small sample sizes that reduced our power to determine statistically meaningful outcomes. Some factors related to missing data included difficulty of the study staff reaching busy public health practitioners who were also part of the COVID-19 response efforts, and loss of staff at several sites due to the “great resignation” during the COVID-19 pandemic and ongoing loss of local public health employees (60, 61). With our study staff responsible for survey data collection, this meant we relied on site staff to let us know when participants completed the program and provide updates on contact information. A final limitation is common to other lifestyle intervention studies where the demographics of those who are most likely to participate (mainly mid-life and older females) limit our generalizability to younger and male adults.

Findings from this study demonstrate the impact our implementation strategies had on the successful scale up of Med-South. Our implementation research to date has been supported by funding from two 5-year research grants. The challenge now is to expand Med-South scale-up across the Southeastern U.S. and sustain it beyond the research funding period. In addition to an effective intervention and implementation strategies, scale-up requires the development of infrastructure to support and sustain going to scale at the regional levels (62). We are beginning to develop this infrastructure within our CDC-funded Prevention Research Center (PRC). The CDC funds PRCs nationwide to engage academic institutions in solving public health problems through community-engaged research (63). As a first step in planning for regional scale-up, our project has created a website housed on the PRC's website to disseminate Med-South materials and online training modules. We are in the process of developing a plan to market these materials through regional organizations with broad reach to HDs and CHCs in the Southeast.

These dissemination efforts will inevitably be insufficient if they are not supported by substantive changes in how public health prevention efforts are funded. CDC is the primary source of funding for state and local health departments, and their funding over the past 2 decades (fiscal year 2014–2023) increased by a mere 6 percent after adjusting for inflation (64, 65). This level of underfunding means the roughly 60 percent of the U.S. adult population living with at least one chronic disease will have limited accessibility and impact of evidence-based public health prevention programs (64, 65). If we are to address this underfunding nationally, substantially more than the 4.4 percent of health spending in 2021 will have to be directed to public health and prevention (65). Increasing public health and prevention funding would support broadscale scale up of programs like Med-South in communities with the greatest health needs (65).

## 5 Conclusion

In summary, we successfully adapted our implementation and delivery approaches for the Med-South lifestyle intervention to fit the context of public health settings during the COVID-19 pandemic. Both implementation and delivery outcomes were positive and staff and participants at CHCs and HDs had highly favorable views of their experience with Med-South. Despite these and other research findings showing scalability and positive health outcomes, statewide scale-up efforts needed for broadscale public health impact will require changes in policies and practices supporting and funding public health services for chronic disease management and support.

## Data Availability

The raw data supporting the conclusions of this article will be made available by the authors, without undue reservation.

## References

[1] Centers for Disease Control and Prevention. Heart Disease Death Rates, Total Population Ages 35+. Available online at: https://www.cdc.gov/dhdsp/maps/national_maps/hd_all.htm (accessed May 20, 2024).

[2] American Diabetes Association. Statistics About Diabetes. Available online at: http://www.diabetes.org/diabetes-basics/statistics/ (accessed May 20, 2024).

[3] Centers for Disease Control and Prevention. National Diabetes Statistics Report. Available online at: https://www.cdc.gov/diabetes/php/data-research/index.html (accessed May 24, 2024).

[4] Centers for Disease Control and Prevention. A Deeper Dive into Diabetes Disparities: An Interactive Module on US Diabetes Statsitics. Available online at: https://gis.cdc.gov/grasp/diabetes/diabetesatlas-disparities.html (accessed May 22, 2024).

[5] Centers for Disease Control and Prevention. Adult Obesity Prevalence Maps. Available online at: https://www.cdc.gov/obesity/php/data-research/adult-obesity-prevalence-maps.html?CDC_AAref_Val=https://www.cdc.gov/obesity/data/prevalence-maps.html (accessed May 18, 2024).

[6] Dwyer-Lindgren L Bertozzi-Villa A Stubbs RW Morozoff C Mackenbach JP van Lenthe FJ. Inequalities in life expectancy among US counties, 1980 to 2014: temporal trends and key drivers. JAMA Intern Med. (2017) 177:1003–11. 10.1001/jamainternmed.2017.0918 28492829 PMC5543324

[7] Okobi OE Ajayi OO Okobi TJ Anaya IC Fasehun OO Diala CS. The burden of obesity in the rural adult population of America. Cureus. (2021) 13:e15770. 10.7759/cureus.15770 34295580 PMC8290986

[8] Bundy JD Mills KT He H LaVeist TA Ferdinand KC Chen J. Social determinants of health and premature death among adults in the USA from 1999 to 2018: a national cohort study. Lancet Public Health. (2023) 8:e422–e31. 10.1016/S2468-2667(23)00081-6 37244672 PMC10349537

[9] Braveman P Gottlieb L. The social determinants of health: it's time to consider the causes of the causes. Public Health Rep. (2014) 129(Suppl. 2):19–31. 10.1177/00333549141291S206 24385661 PMC3863696

[10] Howard G Labarthe DR Hu J Yoon S Howard VJ. Regional differences in African Americans' high risk for stroke: the remarkable burden of stroke for Southern African Americans. Ann Epidemiol. (2007) 17:689–96. 10.1016/j.annepidem.2007.03.019 17719482 PMC1995237

[11] Centers for Disease Control and Prevention. Interactive Atlas of Heart Disease and Stroke. Available online at: https://nccd.cdc.gov/DHDSPAtlas/ (accessed May 18, 2024).

[12] Howard G Howard VJ. Twenty years of progress toward understanding the stroke belt. Stroke. (2020) 51:742–50. 10.1161/STROKEAHA.119.024155 32078485

[13] Dubay LC Lebrun LA. Health, behavior, and health care disparities: disentangling the effects of income and race in the United States. Int J Health Serv. (2012) 42:607–25. 10.2190/HS.42.4.c 23367796

[14] Lee SH Moore LV Park S Harris DM Blanck HM. Adults meeting fruit and vegetable intake recommendations - United States, 2019. MMWR Morb Mortal Wkly Rep. (2022) 71:1–9. 10.15585/mmwr.mm7101a1 34990439 PMC8735562

[15] Blanck HM Gillespie C Kimmons JE Seymour JD Serdula MK. Trends in fruit and vegetable consumption among U.S. men and women, 1994-2005. Prev Chronic Dis. (2008) 5:A35. 18341771 PMC2396974

[16] Centers for Disease Control and Prevention (CDC). Prevalence of self-reported physically active adults–United States, 2007. MMWR Morb Mortal Wkly Rep. (2008) 57:1297–300. 19052527

[17] Krebs-Smith SM Cook A Subar AF Cleveland L Friday J. US adults' fruit and vegetable intakes, 1989 to 1991: a revised baseline for the Healthy People 2000 objective. Am J Public Health. (1995) 85:1623–9. 10.2105/AJPH.85.12.1623 7503335 PMC1615724

[18] Serdula MK Gillespie C Kettel-Khan L Farris R Seymour J Denny C. Trends in fruit and vegetable consumption among adults in the United States: behavioral risk factor surveillance system, 1994-2000. Am J Public Health. (2004) 94:1014–8. 10.2105/AJPH.94.6.1014 15249308 PMC1448382

[19] Cai Y Richards EA. Systematic review of physical activity outcomes of rural lifestyle interventions. West J Nurs Res. (2016) 38:909–27. 10.1177/0193945915625922 26728043

[20] Haskell WL Lee IM Pate RR Powell KE Blair SN Franklin BA. Physical activity and public health: updated recommendation for adults from the American College of Sports Medicine and the American Heart Association. Circulation. (2007) 116:1081–93. 10.1161/CIRCULATIONAHA.107.185649 17671237

[21] Diabetes Prevention Program (DPP) Research Group. The Diabetes Prevention Program (DPP): description of lifestyle intervention. Diabetes Care. (2002) 25:2165–71. 10.2337/diacare.25.12.2165 12453955 PMC1282458

[22] Smith SA Ansa B. A systematic review of lifestyle interventions for chronic diseases in rural communities. J Ga Public Health Assoc. (2016) 5:304–13. 10.21633/jgpha.5.404 27376159 PMC4926769

[23] The Community Guide: Nutrition. Available online at: https://www.thecommunityguide.org/topics/nutrition.html (accessed May 13, 2024).

[24] US Preventive Services Task Force Krist AH Davidson KW Mangione CM Barry MJ Cabana M. Behavioral counseling interventions to promote a healthy diet and physical activity for cardiovascular disease prevention in adults with cardiovascular risk factors: US preventive services task force recommendation statement. JAMA. (2020) 324:2069–75. 10.1001/jama.2020.21749 33231670

[25] Ben Charif A Zomahoun HTV LeBlanc A Langlois L Wolfenden L Yoong SL. Effective strategies for scaling up evidence-based practices in primary care: a systematic review. Implement Sci. (2017) 12:139. 10.1186/s13012-017-0672-y 29166911 PMC5700621

[26] Leeman J Calancie L Hartman MA Escoffery CT Herrmann AK Tague LE. What strategies are used to build practitioners' capacity to implement community-based interventions and are they effective?: a systematic review. Implement Sci. (2015) 10:80. 10.1186/s13012-015-0272-7 26018220 PMC4449971

[27] Keyserling TC Samuel-Hodge CD Pitts SJ Garcia BA Johnston LF Gizlice Z. A community-based lifestyle and weight loss intervention promoting a Mediterranean-style diet pattern evaluated in the stroke belt of North Carolina: the Heart Healthy Lenoir Project. BMC Public Health. (2016) 16:732. 10.1186/s12889-016-3370-9 27495295 PMC4975883

[28] Keyserling TC Sheridan SL Draeger LB Finkelstein EA Gizlice Z Kruger E. A comparison of live counseling with a web-based lifestyle and medication intervention to reduce coronary heart disease risk: a randomized clinical trial. JAMA Intern Med. (2014) 174:1144–57. 10.1001/jamainternmed.2014.1984 24861959 PMC4142754

[29] Samuel-Hodge CD Gizlice Z Allgood SD Bunton AJ Erskine A Leeman J. Strengthening community-clinical linkages to reduce cardiovascular disease risk in rural NC: feasibility phase of the CHANGE study. BMC Public Health. (2020) 20:264. 10.1186/s12889-020-8223-x 32085707 PMC7035725

[30] Samuel-Hodge CD Ziya G Allgood SD Bunton AJ Erskine A Leeman J. Hybrid implementation-effectiveness study of a community health worker-delivered intervention to reduce cardiovascular disease risk in a rural, underserved non-hispanic black population: the CHANGE study. Am J Health Promot. (2022) 36:948–58. 10.1177/08901171221078272 35422132 PMC9198395

[31] Caprara G. Mediterranean-type dietary pattern and physical activity: the winning combination to counteract the rising burden of non-communicable diseases (NCDs). Nutrients. (2021) 13:429. 10.3390/nu13020429 33525638 PMC7910909

[32] US Department of Agriculture and US Department of Health and Human Services. Dietary Guidelines for Americans, 2020-2025. 9th ed. December 2020. DietaryGuidelines.gov.

[33] Barker PM Reid A Schall MW. A framework for scaling up health interventions: lessons from large-scale improvement initiatives in Africa. Implement Sci. (2016) 11:12. 10.1186/s13012-016-0374-x 26821910 PMC4731989

[34] Powell BJ Waltz TJ Chinman MJ Damschroder LJ Smith JL Matthieu MM. A refined compilation of implementation strategies: results from the Expert Recommendations for Implementing Change (ERIC) project. Implement Sci. (2015) 10:21. 10.1186/s13012-015-0209-1 25889199 PMC4328074

[35] Leeman J Draeger LB Lyons K Pham L Samuel-Hodge C. Tailoring implementation strategies for scale-up: preparing to take the Med-South Lifestyle program to scale statewide. Front Health Serv. (2022) 2:934479. 10.3389/frhs.2022.934479 36925769 PMC10012719

[36] NC Rural Center. Rural NC At a Glance. Available online at: https://www.ncruralcenter.org/wp-content/uploads/2023/05/Rural-NC-at-a-Glance.pdf (accessed May 22, 2024).

[37] Curran GM Bauer M Mittman B Pyne JM Stetler C. Effectiveness-implementation hybrid designs: combining elements of clinical effectiveness and implementation research to enhance public health impact. Med Care. (2012) 50:217–26. 10.1097/MLR.0b013e3182408812 22310560 PMC3731143

[38] US Department of Health and Human Services. Physical Activity Guidelines for Americans, 2nd ed. (2018). Available online at: https://health.gov/sites/default/files/2019-09/Physical_Activity_Guidelines_2nd_edition.pdf.

[39] CITI Program. Human Subjects Research (HSR). Available online at: https://about.citiprogram.org/series/human-subjects-research-hsr/ (accessed May 20, 2024).

[40] REDCap (Research Electronic Data Capture). Available online at: https://projectredcap.org/resources/citations/ (accessed May 20, 2024).

[41] Harris PA Taylor R Minor BL Elliott V Fernandez M O'Neal L. The REDCap consortium: building an international community of software platform partners. J Biomed Inform. (2019) 95:103208. 10.1016/j.jbi.2019.103208 31078660 PMC7254481

[42] Harris PA Taylor R Thielke R Payne J Gonzalez N Conde JG. Research electronic data capture (REDCap)–a metadata-driven methodology and workflow process for providing translational research informatics support. J Biomed Inform. (2009) 42:377–81. 10.1016/j.jbi.2008.08.010 18929686 PMC2700030

[43] Proctor E Silmere H Raghavan R Hovmand P Aarons G Bunger A. Outcomes for implementation research: conceptual distinctions, measurement challenges, and research agenda. Adm Policy Ment Health. (2011) 38:65–76. 10.1007/s10488-010-0319-7 20957426 PMC3068522

[44] Block G Gillespie C Rosenbaum EH Jenson C. A rapid food screener to assess fat and fruit and vegetable intake. Am J Prev Med. (2000) 18:284–8. 10.1016/S0749-3797(00)00119-7 10788730

[45] Centers for Disease Control and Prevention. Behavioral Risk Factor Surveillance System 2017 Questionnaire. Available online at: https://www.cdc.gov/brfss/questionnaires/pdf-ques/2017_BRFSS_Pub_Ques_508_tagged.pdf (accessed May 20, 2024).

[46] Kraschnewski JL Gold AD Gizlice Z Johnston LF Garcia BA Samuel-Hodge CD. Development and evaluation of a brief questionnaire to assess dietary fat quality in low-income overweight women in the southern United States. J Nutr Educ Behav. (2013) 45:355–61. 10.1016/j.jneb.2012.10.008 23340242

[47] Jones SA Evenson KR Johnston LF Trost SG Samuel-Hodge C Jewell DA. Psychometric properties of the modified RESIDE physical activity questionnaire among low-income overweight women. J Sci Med Sport. (2015) 18:37–42. 10.1016/j.jsams.2013.12.007 24462117 PMC4184999

[48] Cleland CL Hunter RF Kee F Cupples ME Sallis JF Tully MA. Validity of the global physical activity questionnaire (GPAQ) in assessing levels and change in moderate-vigorous physical activity and sedentary behaviour. BMC Public Health. (2014) 14:1255. 10.1186/1471-2458-14-1255 25492375 PMC4295403

[49] Economic Research Service U. U.S. Household Food Security Survey Module: Six-Item Short Form 2012. Available online at: https://www.ers.usda.gov/media/8282/short2012.pdf (accessed May 20, 2024).

[50] NC Rural Center. County Data. Available online at: https://www.ncruralcenter.org/advocacy-and-research/county-data/ (accessed May 20, 2024).

[51] Bellg AJ Borrelli B Resnick B Hecht J Minicucci DS Ory M. Enhancing treatment fidelity in health behavior change studies: best practices and recommendations from the NIH Behavior Change Consortium. Health Psychol. (2004) 23:443–51. 10.1037/0278-6133.23.5.443 15367063

[52] Kraschnewski JL Keyserling TC Bangdiwala SI Gizlice Z Garcia BA Johnston LF. Optimized probability sampling of study sites to improve generalizability in a multisite intervention trial. Prev Chronic Dis. (2010) 7:A10. 20040225 PMC2811505

[53] AbdulRaheem Y. Unveiling the significance and challenges of integrating prevention levels in healthcare practice. J Prim Care Community Health. (2023) 14:21501319231186500. 10.1177/21501319231186500 37449436 PMC10350749

[54] Parra-Medina D Wilcox S Salinas J Addy C Fore E Poston M. Results of the Heart Healthy and Ethnically Relevant Lifestyle trial: a cardiovascular risk reduction intervention for African American women attending community health centers. Am J Public Health. (2011) 101:1914–21. 10.2105/AJPH.2011.300151 21852629 PMC3222367

[55] Warner ET Glasgow RE Emmons KM Bennett GG Askew S Rosner B. Recruitment and retention of participants in a pragmatic randomized intervention trial at three community health clinics: results and lessons learned. BMC Public Health. (2013) 13:192. 10.1186/1471-2458-13-192 23496916 PMC3599817

[56] Anderson LN Yoshida-Montezuma Y Dewart N Jalil E Khattar J De Rubeis V. Obesity and weight change during the COVID-19 pandemic in children and adults: a systematic review and meta-analysis. Obes Rev. (2023) 24:e13550. 10.1111/obr.13550 36721999

[57] Farrugia F Refalo D Bonello D Cuschieri S. The impact of the COVID-19 pandemic on Mediterranean diet adherence: a narrative systematic review. Nutr Health. (2024) 30:215–33. 10.1177/02601060231187511 37439029 PMC10345400

[58] Freiberg A Schubert M Romero Starke K Hegewald J Seidler A. A rapid review on the influence of COVID-19 lockdown and quarantine measures on modifiable cardiovascular risk factors in the general population. Int J Environ Res Public Health. (2021) 18:8567. 10.3390/ijerph18168567 34444316 PMC8393482

[59] Samuel-Hodge CD Garcia BA Johnston LF Gizlice Z Ni A Cai J. Translation of a behavioral weight loss intervention for mid-life, low-income women in local health departments. Obesity. (2013) 21:1764–73. 10.1002/oby.20317 23408464

[60] Leider JP Castrucci BC Robins M Hare Bork R Fraser MR Savoia E. The exodus of state and local public health employees: separations started before and continued throughout COVID-19. Health Aff. (2023) 42:338–48. 10.1377/hlthaff.2022.01251 36877909

[61] Gittleman M. The “Great Resignation” in Perspective. Monthly Labor Review. Washington, DC: US Bureau of Labor Statistics; Office of Publications and Special Studies (2022). 10.21916/mlr.2022.20

[62] Milat A Lee K Conte K Grunseit A Wolfenden L van Nassau F. Intervention Scalability Assessment Tool: a decision support tool for health policy makers and implementers. Health Res Policy Syst. (2020) 18:1. 10.1186/s12961-019-0494-2 31900230 PMC6942323

[63] Centers for Disease Control and Prevention. Prevention Research Centers. Available online at: https://www.cdc.gov/prc/index.htm (accessed May 24, 2024).

[64] Trust for America's Health. The Impact of Chronic Underfunding on America's Public Health System: Trends, Risks, and Recommendations (2023). Available online at: https://www.tfah.org/wp-content/uploads/2023/06/TFAH-2023-PublicHealthFundingFINALc.pdf (accessed May 24, 2024).

[65] Trust for America's Health. The Prevention and Public Health Fund: Preventing Disease and Reducing Long-Term Health Costs. Available online at: https://www.tfah.org/wp-content/uploads/2023/09/PPHF_Backgrounder_September2023.pdf (accessed May 24, 2024).

[66] R Core Team. R: A Language and Environment for Statistical Computing. Vienna: R Foundation for Statistical Computing (2022). Available online at: https://www.R-project.org/

